# First Record Mutations in the Genes* ASPA* and* ARSA* Causing Leukodystrophy in Jordan

**DOI:** 10.1155/2019/7235914

**Published:** 2019-01-30

**Authors:** Tawfiq Froukh

**Affiliations:** Department of Biotechnology and Genetic Engineering, Philadelphia University, Jerash Road, Amman 11118, Jordan

## Abstract

Leukodystrophies (LDs) are heterogeneous genetic disorders characterized by abnormal white matter in the central nervous system. Some of the LDs are progressive and often fatal. In general, LD is primarily diagnosed based on the neuroimaging; however, definitive diagnosis of the LD type is done using genetic testing such as next-generation sequencing. The aim of this study is to identify the genetic causes of LD in two independent Jordanian cases that exhibit MRI findings confirming LD with no definitive diagnosis using whole exome sequencing (WES). The most likely causative variants were identified. In one case, the homozygous pathogenic variant NM_000049.2:c.914C>A;p.Ala305Glu, which is previously reported in ClinVar, in the gene* ASPA* was identified causing Canavan disease. In the second case, the homozygous novel variant NM_000487.5:c.256C>G;p.Arg86Gly in the gene* ARSA* was identified causing metachromatic leukodystrophy. The two variants segregate in their families. The phenotypes of the two studied cases overlap with assigned diseases. The present study raises the importance of using WES to identify the precise neurodevelopmental diseases in Jordan.

## 1. Introduction

Leukodystrophies (LDs) are a group of heterogeneous inherited metabolic disorders affecting the white matter and caused by defect in the myelin sheet [1]. These disorders occur usually in the first months of life, accompanied by hypotonia, and gradually become spastic diplegia or quadriplegia, developmental delay, seizures, ataxia, and dyskinesia. In the later stages of these disorders, the affected individuals will have trouble in swallowing and breathing and death occurs in most cases [2].

Thirty types of leukodystrophies are described with specific clinical characteristics and genetic causes [3]. The inheritance patterns of the described LD types are autosomal recessive (20 types),* de novo* dominant (8 types), X-linked recessive (1 type), and X-linked dominant (1 type; Table 1). These inheritance patterns strongly suggest LDs as monogenic/Mendelian disorders. The most common types of LDs are metachromatic leukodystrophy, Canavan disease, Krabbe disease, Alexander disease, and X-linked adrenoleukodystrophy [1].

Recognizing the specific type of LDs is challenging because of the limited knowledge about their etiology as all types share white matter signals on brain MRI (the term* leuko* means white and* dystrophy* means wasting). Despite the fact that curative treatment of LDs is currently limited, definitive diagnosis of the LD type is crucial for symptom management and prognostic and genetic counselling [3].

Patients are often presented to the neurologists with concern to LD based on the abnormal neuroimaging. However, several other clinical features alert the clinicians for the possibility of LD including adrenal insufficiency, endocrine disturbances, ophthalmologic abnormalities, cortical visual impairment, hypodontia and oligodontia, dysmorphic facial features, tendinous xanthomas, skeletal impairment, hearing impairment, hepatosplenomegaly, cutaneous abnormalities, ovarian dysgenesis, and gastrointestinal symptoms [3]. If the initial evaluation of the patient indicates the possibility of LD in the patient, then molecular genetic testing is required.

Many genes have been identified that cause myelin defects by genetic linkage analysis or by next-generation sequencing (NGS) [4]. NGS genetic testing using gene panels, whole exome sequencing (WES), or whole genome sequencing (WGS) is advised for patients with clinical suspect of LD. As the number of genes associated with LD is increasing, it is arguable to use NGS as the best genetic testing option [4].

In Jordan, the epidemiological data on LD are very scarce and dozens of LD cases in Jordan are with unknown genetic causes. Therefore, this study aims to identify the genetic causes of LD in two affected girls of two unrelated consanguineous families in Jordan using whole exome sequencing (WES).

## 2. Materials and Methods

### 2.1. Patients

**Figure 1:**
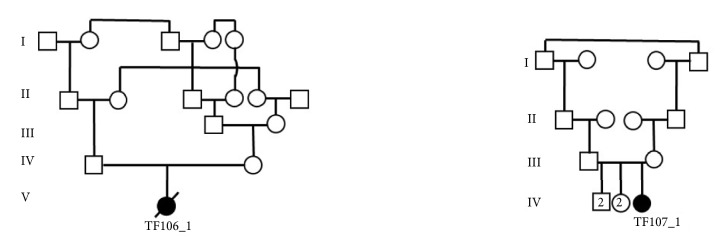
Pedigrees of the studied cases. The dead proband TF106_1 belongs to first-cousin parent once removed. The patient TF107_1 belongs to a first-cousin parent.

Two patients (TF106_1 and TF107_1) were enrolled in this study at the Department of Biotechnology and Genetic Engineering at Philadelphia University in Jordan. The two patients were clinically diagnosed with LD based on the brain MRI. The two patients are unrelated girls; TF106_1 belongs to first-cousin parents once removed, and TF107_1 belongs to first-cousin parents (Figure 1). The clinical characteristics of the two girls were collected from the clinician reports, MRI findings, and the developmental delay as reported by the parents. The girls were two years old by the time of enrolling this study. The study was conducted with the patient's families understanding and informed consent was obtained from the two families.

Blood samples were collected from the two patients, their parents, and their healthy siblings. Total Genomic DNA was extracted according to the standard protocols of the Qiagen FlexiGene DNA kit.

### 2.2. Whole Exome Sequencing (WES)

Because the majority of monogenic diseases can be detected in the coding part of the genome, the DNA samples of the two patients underwent WES using Illumina NOVASEQ6000 platform (Illumina Inc., San Diego, CA, USA). Exomes were captured using SureSelectXT Library Prep Kit (Agilent Technologies, USA). The sequencing reads (150 bp pair end) were mapped to the reference genome (UCSC hg19) using the Burrows-Wheeler Aligner software [5]. Polymerase chain reaction duplicates were removed using samblaster [6]. Single-nucleotide variants and small insertions/deletions (indels) were called using freebayes [7] and annotated using SnpEff-3.3 (Ensembl-GRCh37.73) [8]. Sequencing was conducted by Macrogen (Seoul, Republic of Korea) and the pipeline megSAP was used [9].

To identify possible disease-causing mutations, all high-quality variants were identified that are located in the protein coding region (according to Ensembl database v68) and/or two base pair flanking splice sites. We maintained only the variants meeting the following quality criteria: (1) at least 10X coverage and (2) mapping quality score ≥60.

### 2.3. Copy Number Variant (CNV)

CNV deletions and duplications were checked using the whole exome sequence (WES) coverage data based on the used WES pipeline (megSAP; https://github.com/imgag/megSAP).

### 2.4. Variants Filtration

Rare homozygous variants with minor allele frequency (MAF) ≤ 0.01 were maintained. Filtration was then based on predicted effects of the variants on the protein, maintaining only loss of function (LOF) variants (stop gain, frameshift, splice site acceptor, and splice site donor) and nonsynonymous variants predicted to be probably damaging or possibly damaging by Polyphen2 Humvar [10]. The putative functional homozygous variants were then filtered out if the same variants were observed in a homozygous state in one of the following databases: exome aggregation consortium (ExAC), genome aggregation database (gnomAD), exome variant server (EVS), or the in-house sequenced controls (individuals sequenced as part of other genetic studies at Philadelphia University). LOF variants were also excluded if the gene they are located in carries other homozygous LOF variants in EXAC, gnomAD, EVS, or in-house controls. Variants were then prioritized based on (1) the variants pathogenicity as stated in ClinVar and/or HGMD (The Human Gene Mutation Database) especially for LD disorders, (2) gene/protein function, (3) gene expression profile, (4) effect of gene mutations in mice, and (5) variant segregation in the family. All candidate variants were inspected visually using the software Integrative Genomic Viewer (IGV) [11, 12].

### 2.5. Sanger Sequencing

Sanger sequencing was performed to segregate and confirm the candidate variants. Primers were designed using primer3 version 4.1.0 [13, 14]. Target fragments were amplified using Taq polymerase (Invitrogen), purified with ExoSAP-IT (Affymetrix Inc.), and sequenced using BigDye™ Terminator V.3.1 cycle sequencing kit and ABI PRISM 3730XL sequencer (Applied Biosystems Inc., USA). Sequences were aligned and analysed using Chromas Lite 2.1.1 (Australia Technelysium Pty Ltd).

## 3. Results and Discussion

### 3.1. Clinical Findings

Besides the LD, as reported in the MRI findings (not shown) of the two patients, patient TF106_1 exhibits atonia in her muscles (neck, arms, and legs), blindness, and seizure. The patient died by the age of 23 months. Patient TF107_1 exhibits the following phenotypes progressing slowly: muscle weakness, unsteady gait, and mental deterioration.

### 3.2. Genetic Findings

#### 3.2.1. CNV

CNV deletions and duplications were checked first based on the WES coverage data and no significant deletions or duplications were detected in both patients.

#### 3.2.2. Patient TF106_1

**Figure 2:**
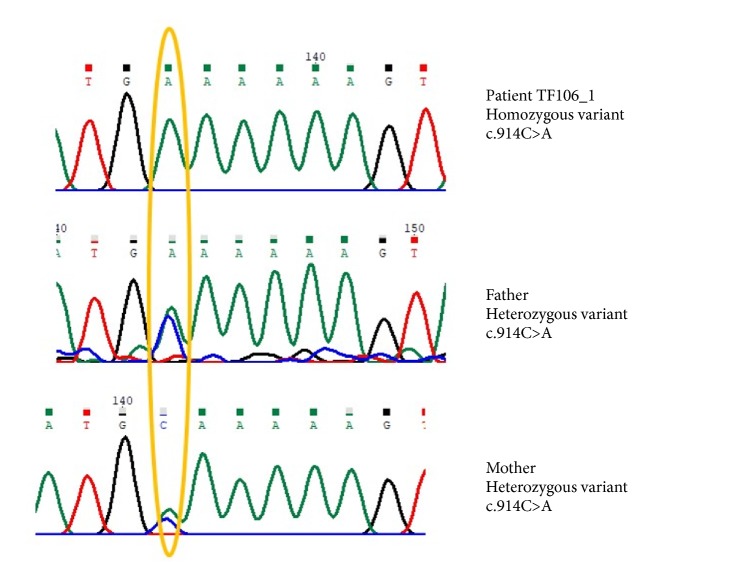
Segregation analysis of the identified variant in the patient TF106_1.

The total number of variants that were revealed after the filtration procedure was 6. The prioritized variant is NM_000049.2:c.914C>A;p.Ala305Glu in the gene* ASPA*, because it is reported as pathogenic in ClinVar (variant ID 2607; Allele ID 17646). The variant is homozygous in the patient and heterozygous in the parents (Figure 2). Pathogenic mutations in the gene ASPA cause Canavan disease (OMIM#271900). Mutations in the gene* ASPA* lead to deficiency of the enzyme aspartoacylase which hydrolyzes N-acetyl-L-aspartic acid (NAA) to aspartate and acetate leading to accumulation of NAA [15]. It is worth mentioning that the annotated exome data file does not harbour any other pathogenic variant for any of the other LD disorders.

Canavan disease is neurodegenerative disease that belongs to LDs. And debate is still for the pathophysiology between the accumulation of NAA and demyelination [16]. The phenotypes of the patient show significant overlap with the clinical synopsis of Canavan disease including increasing head circumference, deafness, blindness, hypotonia, spasticity, seizure, demyelination in the white matter, and death within the first two years of life [17]. Based on these findings, the identified variant in the gene* ASPA* is assigned as causative for the patient phenotype.

#### 3.2.3. Patient TF107_1

**Figure 3:**
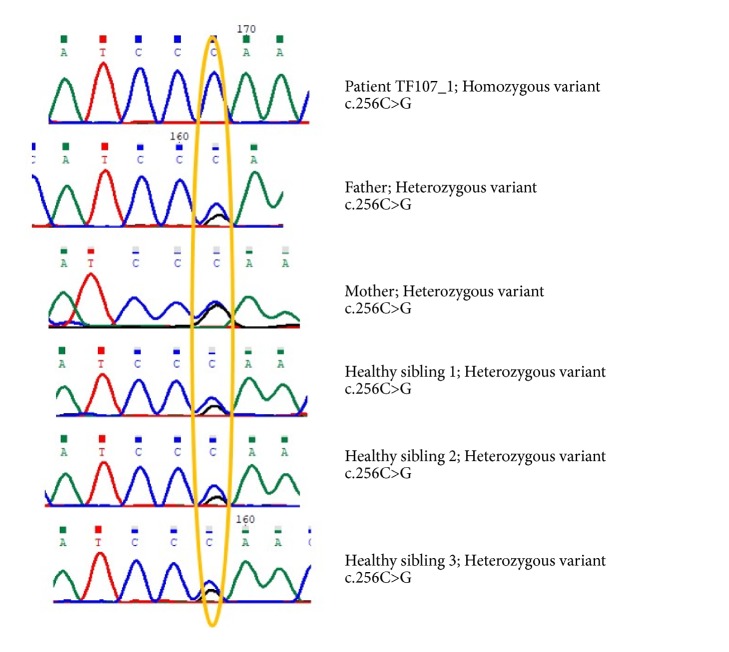
Segregation analysis of the identified variant in the patient TF107_1.

Nine variants were revealed after the filtration procedure. One variant NM_000487.5:c.256C>G;p.Arg86Gly in the gene* ARSA* is associated with OMIM disease. The variant is homozygous in the patient and heterozygous in the parents in all healthy siblings (Figure 3). Homozygous pathogenic variants in the gene* ARSA* cause metachromatic leukodystrophy (MLD) (OMIM#250100). The mutation in* ARSA* gene leads to deficiency in the gene producing the lysosomal enzyme arylsulfatase A which hydrolyzes cerebroside sulfate to cerebroside and sulfate [18]. It is worth mentioning that the annotated exome data file does not harbour any other pathogenic variant for any of the other LD disorders.

The phenotypes of the patient show significant overlap with the clinical synopsis of metachromatic leukodystrophy including mental deterioration, loss of speech, hypotonia, muscle weakness, gait disturbances, ataxia, and demyelination [19]. Based on these findings, the identified variant is assigned as causative for the patient phenotypes. This variant is novel in the gene* ARSA*.

## 4. Conclusion

In summary, the studied patients were genetically diagnosed with Canavan disease and metachromatic leukodystrophy. The two diseases are caused by the lack of important enzymes and thus result in LD. The clinicians in Jordan diagnosed the two independent causes with LD without definitive diagnosis to the type of LD. Here, with the aid of next-generation sequencing (NGS) the genetic causes of the two diseases were assigned. This will further help the two families to avoid having more children with similar diseases and will alert the carrier healthy siblings for their future. Together with the few studies that were conducted in Jordan to identify the genetic causes of neurodevelopmental diseases [20–23], the results of this study stress the need to combine the clinical description of the patients with genetic testing including NGS.

## Figures and Tables

**Table 1:** List of the LD diseases and the associated inheritance patterns.

LD Diseases^1^	Inheritance pattern^2^
Pol-III related disorders (4H syndrome (hypomyelination, hypodontia and hypogonadotropic hypogonadism))	AR
18q minus syndrome	AD
X linked Adrenoleukodystrophy	XLD
Adult onset leukodystrophy with neuroaxonal spheroids and pigmented glia (including hereditary diffuse leukoencephalopathy with spheroids, HDLS, and pigmentary type of orthochromatic leukodystrophy with pigmented glia, POLD)	AD
Aicardi–Goutières Syndrome	AD
Alexander disease	AD
Autosomal Dominant Leukodystrophy with Autonomic disease	AD
Canavan disease	AR
Cerebrotendinous Xanthomatosis	AR
Chloride Ion Channel 2 (ClC-2) related leukoencephalopathy with intramyelinic oedema	AR
eIF2B related disorder (Vanishing White Matter Disease or Childhood ataxia with central nervous system hypomyelination (CACH))	AR
Fucosidosis	AR
Globoid cell Leukodystrophy (Krabbe)	AR
Hypomyelination with atrophy of the basal ganglia and cerebellum (H-ABC)	AD
Hypomyelination with brainstem and spinal cord involvement and leg spasticity (HBSL)	AR
Hypomyelination with congenital cataract (HCC)	AR
Leukoencephalopathy with brainstem and spinal cord involvement and lactate elevation (LBSL)	AR
Leukoencephalopathy with thalamus and brainstem involvement and high lactate (LTBL)	AR
Megalencephalic Leukoencephalopathy with subcortical cysts (MLC)	AR
Metachromatic leukodystrophy (MLD) and its biochemical variants	AR
Oculodentodigital dysplasia	AD
Pelizaeus Merzbacher disease (PMD)	XLR
Pelizaeus Merzbacher like-disease (PMLD)	AR
Peroxisomal Biogenesis disorders (including Zellweger, neonatal Adrenoleukodystrophy and Infantile Refsum)	AR
Polyglucosan Body Disease (PGBD)	AR
RNAse T2 deficient leukoencephalopathy	AR
Sialic acid storage disorders (Salla disease, Infantile Sialic Acid Storage Disease and Intermediate form)	AR
Single enzyme deficiencies of peroxisomal fatty acid beta oxidation (including only D-Bifunctional Protein Deficiency; Sterol Carrier Protein X (SCPx) deficiency; Peroxisomal acyl-CoA-Oxidase Deficiency)	AR
Sjögren–Larsson syndrome	AR
SOX10-associated PCWH: peripheral demyelinating neuropathy, central dysmyelinating leukodystrophy, Waardenburg syndrome, and Hirschsprung disease	AD

^1^LD diseases as indicated in [3].

^2^Inheritance pattern as stated in OMIM database (www.omim.org); AR: autosomal recessive; AD: autosomal dominant; XLD: X-linked dominant; XLR: X-linked recessive.

## Data Availability

The data used to support the findings of this study are included within the article.
